# MEKK1-MKK4-JNK-AP1 Pathway Negatively Regulates Rgs4 Expression in Colonic Smooth Muscle Cells

**DOI:** 10.1371/journal.pone.0035646

**Published:** 2012-04-24

**Authors:** Yonggang Zhang, Fang Li, Shu Liu, Hong Wang, Sunila Mahavadi, Karnam S. Murthy, Kamel Khalili, Wenhui Hu

**Affiliations:** 1 Department of Neuroscience, Temple University School of Medicine, Philadelphia, Pennsylvania, United States of America; 2 Department of Physiology and Biophysics, Medical College of Virginia Campus, Virginia Commonwealth University, Richmond, Virginia, United States of America; University of Louisville, United States of America

## Abstract

**Background:**

*R*egulator of *G*-protein *S*ignaling *4* (RGS4) plays an important role in regulating smooth muscle contraction, cardiac development, neural plasticity and psychiatric disorder. However, the underlying regulatory mechanisms remain elusive. Our recent studies have shown that upregulation of Rgs4 by interleukin (IL)-1β is mediated by the activation of NFκB signaling and modulated by extracellular signal-regulated kinases, p38 mitogen-activated protein kinase, and phosphoinositide-3 kinase. Here we investigate the effect of the c-Jun N-terminal kinase (JNK) pathway on Rgs4 expression in rabbit colonic smooth muscle cells.

**Methodology/Principal Findings:**

Cultured cells at first passage were treated with or without IL-1β (10 ng/ml) in the presence or absence of the selective JNK inhibitor (SP600125) or JNK small hairpin RNA (shRNA). The expression levels of *Rgs4* mRNA and protein were determined by real-time RT-PCR and Western blot respectively. SP600125 or JNK shRNA increased Rgs4 expression in the absence or presence of IL-1β stimulation. Overexpression of MEKK1, the key upstream kinase of JNK, inhibited Rgs4 expression, which was reversed by co-expression of JNK shRNA or dominant-negative mutants for MKK4 or JNK. Both constitutive and inducible upregulation of Rgs4 expression by SP600125 was significantly inhibited by pretreatment with the transcription inhibitor, actinomycin D. Dual reporter assay showed that pretreatment with SP600125 sensitized the promoter activity of *Rgs4* in response to IL-1β. Mutation of the AP1-binding site within *Rgs4* promoter increased the promoter activity. Western blot analysis confirmed that IL-1β treatment increased the phosphorylation of JNK, ATF-2 and c-Jun. Gel shift and chromatin immunoprecipitation assays validated that IL-1β increased the *in vitro* and *ex vivo* binding activities of AP1 within rabbit *Rgs4* promoter.

**Conclusion/Significance:**

Activation of MEKK1-MKK4-JNK-AP1 signal pathway plays a tonic inhibitory role in regulating *Rgs4* transcription in rabbit colonic smooth muscle cells. This negative regulation may aid in maintaining the transient level of RGS4 expression.

## Introduction

Signal transduction is a key process of converting one signal to another, leading to a series of signaling reactions. One critical class of signal-transduction pathways is the signaling controlled by the guanine–nucleotide-binding heterotrimeric proteins (G proteins). G protein-coupled receptors (GPCRs), also known as seven-transmembrane domain receptors, comprise a large protein family of transmembrane receptors. GPCRs are involved in a vast array of physiological and pathological processes and are also the targets of approximately 40% of all modern medicinal drugs [1], [2]. The ligand binding to GPCRs, such as the acetylcholine (ACh) receptor, catalyzes GDP-GTP exchange on the α-subunit of a heterotrimeric G-protein complex. The dissociation of GTP-bound α-subunit from βγ subunits leads to the regulation of downstream effectors. GPCR signaling is terminated by the intrinsic GTPase activity of the Gα-subunit, which is accelerated by the regulator of G-protein signaling (RGS) proteins as GTPase-activating proteins. RGS proteins regulate the strength and duration of Gα signaling [2]. Each RGS protein regulates the function of multiple GPCRs, while some RGS proteins have a clear preference for particular receptor-G protein complexes. RGS4 is one of seven members of a classic R4 RGS protein family that accelerates the intrinsic GTPase activity of the Gαi/o and Gαq/11 family members [3]. RGS4 plays an important role in regulating smooth muscle contraction, cardiomyocyte development, neural plasticity and psychiatric disorders [4]–[7]. In particular, RGS4 has been widely shown to be an underlying risk factor for schizophrenia, even though it is not true in some human populations [4], [8]–[12].

In neuronal cell lines, expression of Rgs4 is reduced after treatment with nerve growth factor [13], cAMP [14] or camptothecin [15], whereas opioid receptor agonists lead to an increase in the expression levels of *Rgs4* mRNA [16] and RGS4 protein [17]. Administration of corticosterone to adult rats decreases the level of *Rgs4* mRNA in the paraventricular nucleus of the hypothalamus and increases the levels in locus coeruleus [18], but has no effect in the hippocampus [19], [20]. Long-term opiate administration is associated with an increase in RGS4 immunoreactivity in the rat and human brain [21], [22]. Rapid kindling leads to an increase of *Rgs4* mRNA in hippocampus and forebrain, but not in brainstem or cerebellum [23]. Rgs4 expression is downregulated in prefrontal cortex and striatum by neonatal status epilepticus [24]. In rat adrenal glands, Rgs4 is upregulated by aldosterone secretagogues, both *in vivo* and *in vitro* [25]. *Rgs4* mRNA is expressed only in glial cell line-derived neurotrophic factor-responsive neurons [26]. In cardiomyocyte, Rgs4 expression is induced by endotoxin and interleukin (IL)-1β [27], [28] and may contribute to the loss of Gα_q_-mediated phospholipase C activation by endothelin-1 [29]. In human aortic smooth muscle cells (SMC), *RGS4* is highly expressed at the mRNA level and inhibits S1P_3_ receptor-mediated signaling [30]. In gastrointestinal smooth muscle, Rgs4 negatively regulates Gα_q_ signaling activated by M3 or motilin receptors [31], [32] and thus inhibits agonist-induced initial contraction [6], [7], [33]. In our previous studies, we demonstrated for the first time that Rgs4 expression is increased in both dispersed and cultured rabbit SMC after IL-1β treatment [7]. These findings suggest that Rgs4 expression is regulated dynamically by inflammatory mediators such as cytokines and growth factors.

However, the molecular mechanisms and signaling pathways for RGS4 regulation remain elusive. At the protein level, Rgs4 is regulated by the N-end rule pathway [34], [35] and proteasome degradation [6], [36]. At the mRNA level, *Rgs4* is regulated by a transcription factor Phox2b [37]. Our recent studies demonstrate that IL-1β-induced upregulation of Rgs4 is transcription-dependent [6], [38] and mediated by the canonical IKK2/IκBα pathway of NFκB activation [6]. Further studies suggest that IL-1β-induced activation of either extracellular signal-regulated kinase 1/2 (ERK1/2) or p38 mitogen-activated protein (MAP) kinase (MAPK) enhances the upregulation of Rgs4 expression, whereas the PI3K/Akt/GSK3β pathway attenuates IL-1β-induced upregulation of Rgs4 expression [39].

The pathway of c-Jun NH2-terminal kinase (JNK), also known as stress-activated protein kinase, is another key member of MAPK superfamily, and is activated primarily by inflammatory cytokines and environmental stresses [40]–[42]. The JNK family includes JNK1 (four isoforms), JNK2 (four isoforms), and JNK3 (two isoforms). JNKs are activated by MAP2kinases such as MAPK kinase (MKK)4 and MKK7, which are in turn activated by the MAP3kinases, such as MAP-ERK kinase kinase (MEKK)1, MEKK4, TAK1, ASK1 and MLKs [43]. The JNK MAP3kinase pathways are activated by MAP4kinases that link to a variety of cell receptors [40], [44]. The diversity and selection of upstream kinases for JNK activation depend upon the cell types and stimulators [40]. After activation, JNK regulates target gene expression through an array of transcription factors such as AP1, ATF-2, SMAD4, NFAT, etc. [45]–[47]. In the present study, we investigated the role of MEKK1-MKK4-JNK-AP1 pathway in regulating Rgs4 expression in rabbit colonic SMC and showed that JNK inhibition increased while MEKK1/MKK4 overexpression attenuated both constitutive and IL-1β-induced expression of Rgs4. IL-1β induced transient phosphorylation of JNK and sustained phosphorylation of c-Jun and ATF-2. IL-1β increased the binding activity of c-Fos and c-Jun to *Rgs4* promoter. JNK inhibition and mutation of the AP1-binding site within the *Rgs4* promoter sensitized the promoter activity of *Rgs4* in response to IL-1β. This work provides new insights into how stress-induced signaling pathways regulate G protein signaling and smooth muscle contraction.

## Results

### Pharmacological inhibition of JNK by SP600125 significantly increased Rgs4 expression in colonic SMC

**Figure 1:**
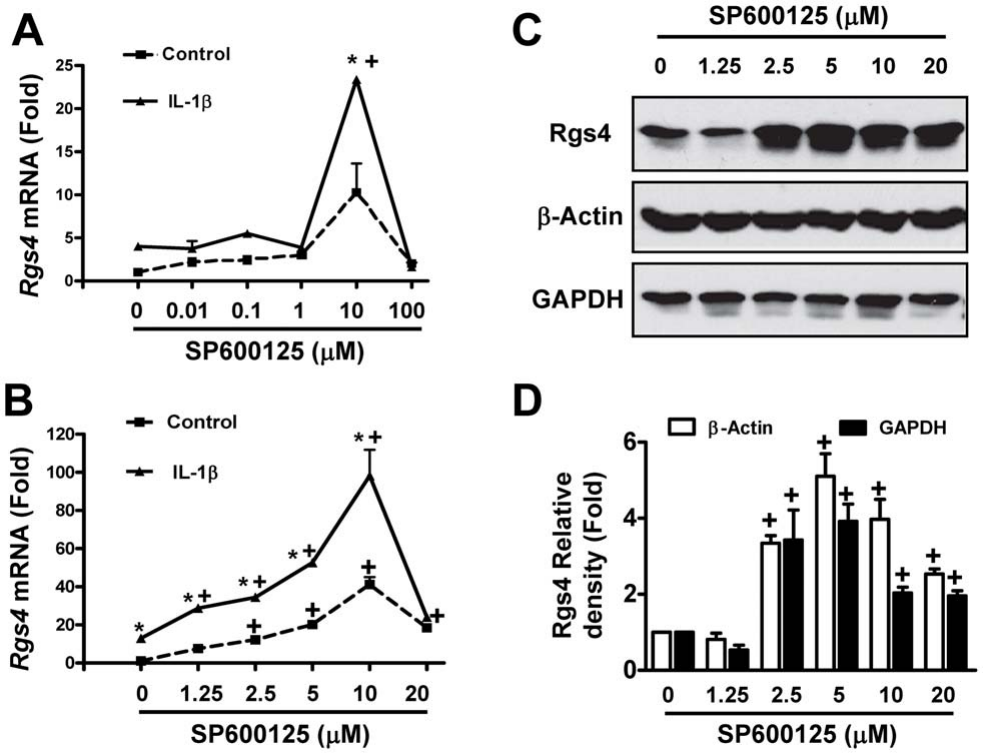
The JNK inhibitor SP600125 dose-dependently increases Rgs4 expression in rabbit colonic smooth muscle cells. Cultured and serum-starved muscle cells were treated with indicated concentration of SP600125 1 h before treatment with IL-1β (10 ng/ml) for 3 h, followed by reverse transcriptase quantitative polymerase chain reaction (RT-qPCR) (A, B) and Western blot analysis (C, D). The relative level of *Rgs4* mRNA expression (fold induction) was presented as compared with the control without SP600125 pretreatment after GAPDH normalization (A, B). Levels of β-actin and GAPDH were used as a loading control (C). The relative optical density (fold change) was presented as compared with the vehicle control (DMSO) after β-actin or GAPDH normalization (D). Values are means ± SE of 3 experiments. * (p<0.05) indicates significant increase after IL-1β treatment as compared with the control. + (p<0.05) indicate significant increase by ANOVA and Newman-Keuls comparison of SP600125 treatment with the vehicle control.

IL-1β is well known to activate NFκB and MAPK pathways [48]–[50]. We have shown that the NFκB pathway, as well as the ERK1/2 and p38 MAPK pathways enhance while the PI3K/Akt/GSK3β pathway inhibits the upregulation of Rgs4 expression by IL-1β in colonic SMC [6], [39]. To explore the potential role of JNK pathway on Rgs4 expression in colonic SMC, we examined the effect of JNK pathway inhibitor on Rgs4 expression by reverse transcription-quantitative polymerase chain reaction (RT-qPCR) and Western blot analysis, the established techniques for detecting *Rgs4* mRNA and protein expression [7]. SP600125, a well-established specific inhibitor for the JNK pathway [48], [50], [51], were selected to pretreat the serum-starved SMC for 1 h at different concentrations before IL-1β (10 ng/ml) stimulation for 3 h. Total RNA extraction and whole cell lysate were prepared. In the preliminary studies, a long range concentration (10 nM to 100 µM) of SP600125 was tested, showing the maximal effect at 10 µM (Fig. 1A). Thus, 1–20 µM was used for the present study. SP600125 treatment alone between 1–10 µM induced a dose-dependent upregulation of *Rgs4* mRNA (Fig. 1B) and protein (Fig. 1C). However, a higher concentration of SP600125 (20 µM) did not induce further upregulation but reversed the expression of *Rgs4* mRNA and protein (Fig. 1B–D), which may result from the non-specific effects on other kinases. IL-1β treatment alone increased *Rgs4* mRNA expression as previously reported [6], [7]. Pretreatment with SP600125 enhanced IL-1β-induced upregulation of *Rgs4* mRNA in a dose-dependent manner similar to SP600125 alone (Fig. 1B). However, SP600125 at 20 µM did not induce additive or an increased effect over IL-1β, perhaps due to additional toxic effect. These data suggest that inhibition of the JNK pathway enhances constitutive and IL-1β-induced expression of Rgs4 in colonic SMC. Therefore, 10 µM of SP600125, consistent with previous reports [48], [50]–[52], was used for further functional and mechanistic studies.

### Knockdown of JNK expression by shRNA increased Rgs4 expression in colonic SMC

**Figure 2:**
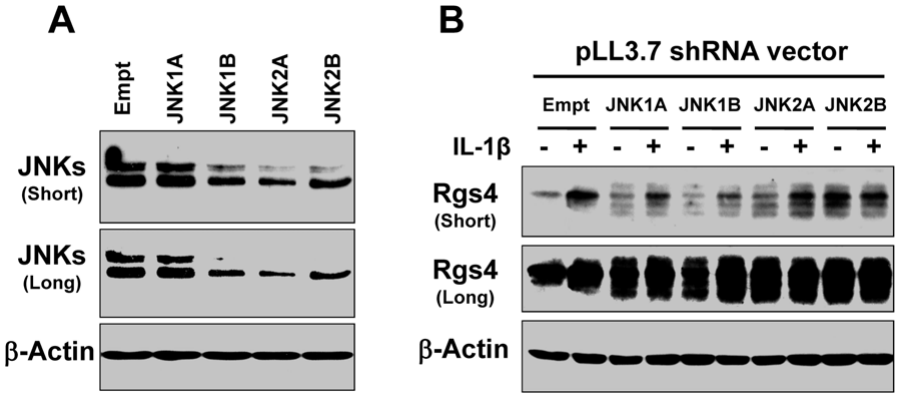
Knockdown of JNK protein expression by shRNA (A) increased Rgs4 protein expression (B) in rabbit colonic smooth muscle cells. Cultured cells were transfected with pLL3.7 empty vector or indicated JNK shRNA expression vectors. After 48 h, cells were starved for 24 h and treated with vehicle control or IL-1β (10 ng/ml) for 3 h, followed by Western blotting with anti-JNK (A) or anti-RGS4 (B) antibodies. The β-actin was used for loading control. Short and long exposures of the blot are shown. Similar results were observed in 3 experiments.

To validate the stimulatory effect of JNK pharmacologic inhibition, we tested the effect of JNK specific shRNA silencing on constitutive and IL-1β-induced Rgs4 expression. The efficacy of JNK1 and JNK2 shRNA was validated by Western blot analysis (Fig. 2A) with anti-JNK(FL) antibody, which recognized p46 and p54 isoforms of JNK1, JNK2 and JNK3 (manufacture's data sheet). The p46 isoforms contain JNK1a1, JNK1b1, JNK2a1, JNK2b1, and JNK3a1, while the p54 isoforms contain JNK1a2, JNK1b2, JNK2a2, JNK2b2, and JNK3b2 [53]. As shown in Fig. 2B, both JNK1 and JNK2 shRNA dramatically increased the constitutive and IL-1β-induced expression of Rgs4 protein, and the effect of JNK2 shRNA was stronger than that of JNK1 shRNA (Fig. 2A). Consistent with SP600125 (Fig. 1C), both JNK1 and JNK2 shRNA increased the number of bands detected by Rgs4 antibody, implying that JNK may regulate the protein stability of Rgs4 [6], [34]–[36].

### The effects of JNK inhibition on the constitutive and IL-1β-induced expression of Rgs4 mRNA were transcription-dependent

**Figure 3:**
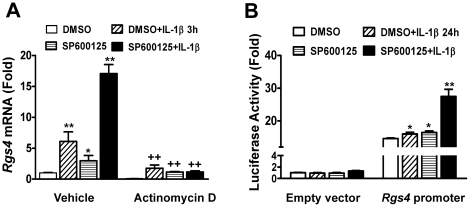
Inhibition of JNK pathway potentiates *Rgs4* transcription in rabbit colonic smooth muscle cells. ***A. Transcriptional inhibition prevents Rgs4 mRNA upregulation by IL-1β and SP600125.*** Cultured muscle cells were starved for 24 h and pretreated with actinomycin D (10 µM) for 1 h and SP600125 (10 µM) for 30 min before exposure to IL-1β (10 ng/ml) for 3 h. Expression level (fold change) of *Rgs4* mRNA was determined by RT-qPCR using GAPDH for normalization. ***B. SP600125 potentiates constitutive and IL-1β-induced promoter activity of rabbit Rgs4.*** Cultured muscle cells were cotransfected with promoter-less pMlu3 empty vector or *Rgs4* promoter vector carrying secreted *renilla* luciferase and pGL4-CMV vector carrying *firefly* luciferase (for normalization). After 24 h, cells were serum-starved for 24 h and treated with IL-1β (10 ng/ml) and SP600125 (10 µM) for 24 h. The *renilla* and *firefly* luciferases were measured separately. The relative fold changes in *renilla* luciferase activity after normalization by *firefly* luciferase were expressed as compared with the empty vector and vehicle DMSO treatment. Data represents the mean ± SEM of 4 experiments, each with quadruplicate. ** P<0.01 and * P<0.05 indicate statistically significant increase by student's *t* test compared with corresponding DMSO treatment. ^++^ (p<0.01) indicates significant decrease after actinomycin D treatment compared with corresponding vehicle control.

To investigate whether the transcriptional mechanism is involved in the enhancing effect of JNK inhibition on *Rgs4* mRNA expression, cultured SMC were pretreated with the transcription inhibitor, actinomycin D (10 µM) 1 h before SP600125 (10 µM) was applied for 4 h and IL-1β for 3 h. The level of *Rgs4* mRNA expression was determined by RT-qPCR and normalized to the house-keeping gene GAPDH. Consistent with previous studies [6], pretreatment with actinomycin D blocked IL-1β-induced upregulation of *Rgs4* mRNA expression (Fig. 3A). Actinomycin D pretreatment completely blocked the upregulation of *Rgs4* mRNA induced by either SP600125 alone or a combination of SP600125 and IL-1β (Fig. 3A). These data suggest that inhibition of the JNK pathway stimulates the transcription of Rgs4 in colonic SMC.

To further understand the transcriptional mechanism underlying the induction of *Rgs4* mRNA expression by JNK inhibition, we performed a luciferase reporter assay for *Rgs4* promoter activity by transfecting SMC with rabbit *Rgs4* promoter-luciferase reporter plasmid [38]. As shown in Fig. 3B, inhibition of JNK with SP600125 alone significantly increased the promoter activity of *Rgs4* in a similar manner to the effect of IL-1β stimulation. Pretreatment with SP600125 before IL-1β exposure sensitized the promoter activity of *Rgs4* in response to IL-1β (Fig. 3B). These data suggest that activation of endogenous JNK pathway plays a tonic inhibitory effect on the constitutive and IL-1β-inducible promoter (transcription) activity of *Rgs4*.

### JNK-AP1 pathway maintained a tonic inhibition of Rgs4 transcription

**Figure 4:**
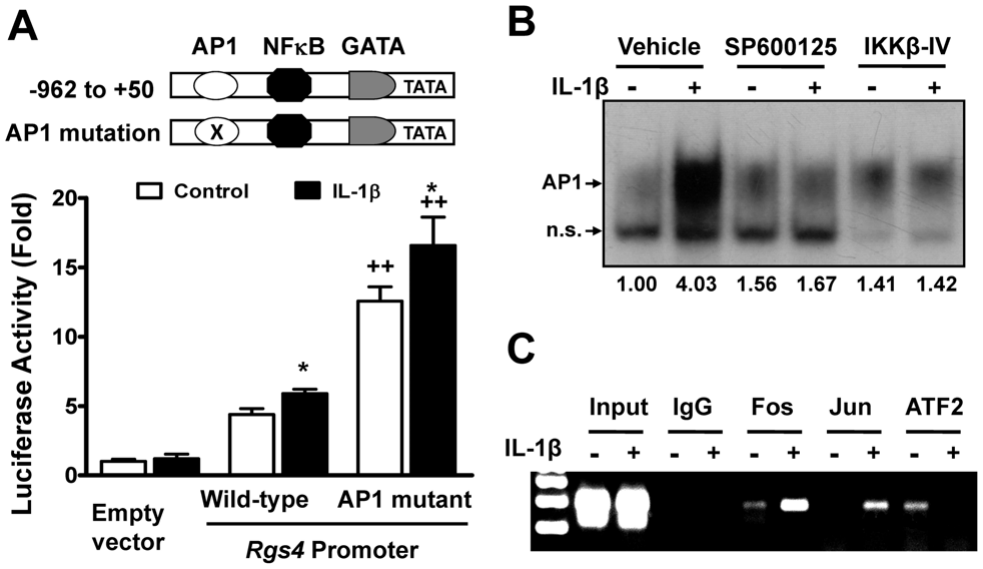
IL-1β promoted AP1-binding activity within proximal *Rgs4* promoter. *A. Inhibitory function of AP1 binding site within Rgs4 promoter for activation of reporter gene.* Site-directed mutant of AP1 site from Rgs4 promoter was co-transfected with normalization vector into cultured smooth muscle cells. After 24 h, cells were serum-starved for 24 h and treated with IL-1β (10 ng/ml) for 24 h before relative luciferase activity was determined. * P<0.05 indicates a statistically significant increase by student's *t* test compared with control treatment. ^++^ P<0.01 indicates a significant increase by AP1 site mutation in promoter activity compared with the wild-type *Rgs4* promoter. Values represent the mean ± SEM of 4 individual experiments. ***B. Induction of AP1-DNA binding activity by IL-1β that is blocked by JNK or NFκB inhibition.*** Serum-starved muscle cells were treated with or without IL-1β (10 ng/ml) for 3 h in the absence or presence of JNK inhibitor SP600125 (10 µM) or NFκB inhibitor IKK2-IV (10 µM), and nuclear extracts were prepared for electrophoretic mobility shift assay using AP1-binding motif of rabbit *Rgs4* promoter. The number under each panel indicates the relative fold of optical density compared with the corresponding control. n.s. for non-specific band. ***C. Induction of endogenous c-Fos- and c-Jun-DNA binding activity but inhibition of ATF-2-DNA binding within Rgs4 promoter by IL-1β.*** Serum-starved muscle cells were treated with IL-1β for 3 h before chromatin immunoprecipitation assay with indicated antibodies. Input indicates the DNA from supernatant after precipitation without IgG. Data are representative of 3 experiments.

The family of AP1 transcription factor consists of several subfamilies of bZIP-domain (bZIP = basic region leucine zipper) proteins: the Jun (c-Jun, JunB, and JunD), the Fos (c-Fos, FosB, Fra-1 and Fra-2), and the ATF-2 (ATF-2 and ATF-a) [54]. Since AP1 is a major target of the JNK signaling pathway, and an AP1 binding site within the proximal region of rabbit *Rgs4* promoter was identified by bioinformatics analysis using MatInspector [38], we hypothesize that the JNK pathway inhibits *Rgs4* transcription predominantly via AP1 transcription factor. To test this hypothesis, we first examined the function of AP1 binding site within *Rgs4* promoter using *Rgs4* promoter luciferase reporter assay and site-directed mutagenic analysis. As shown in Fig. 4A, mutation of the AP1-binding site within rabbit *Rgs4* promoter increased the promoter activity and sensitized IL-1β-induced promoter activity. These data imply that the AP1 binding site is required for the tonic inhibitory effect of the JNK pathway activation on *Rgs4* transcription and the transcription factor AP1 functions as a repressor for *Rgs4* regulation.

We then determined if IL-1β treatment affects the binding activity of AP1 transcription factor within the *Rgs4* promoter both *in vitro* and *ex vivo*. Electrophoretic mobility shift assay (EMSA) measuring the *in vitro* interactions between an oligonucleotide probe containing rabbit *Rgs4* attgagtcact sequence and SMC nuclear protein showed that IL-1β induced the formation of an AP1 DNA-binding complex, which was completely blocked by the specific inhibitor of either JNK pathway or NFκB pathway (Fig. 4B). The *ex vivo* chromatin immunoprecipitation (CHIP) assay on the chromatin of cultured rabbit colonic SMC identified a specific enrichment of AP1 transcription factor within proximal *Rgs4* promoter containing the AP1 binding site by CHIP assay with antibodies against c-Fos, c-Jun and ATF-2, the key components of AP1 transcription factor (Fig. 4C). The epitope-matching control IgG was used as a negative control for CHIP and the input chromatin DNA was used as a positive control for PCR. In non-stimulated cells, both c-Fos and ATF-2 were found to bind to *Rgs4* promoter but c-Jun was absent (Fig. 4C). IL-1β treatment for 3 h promoted the DNA-binding activity of endogenous c-Fos and c-Jun proteins but removed ATF-2 from the *Rgs4* promoter (Fig. 4C). These data suggest that IL-1β promoted DNA-binding activity of Fos/Jun-containing AP1 factors within proximal *Rgs4* promoter that ultimately suppressed the transcription of *Rgs4*.

### IL1-β induced rapid activation of the JNK-AP1 pathway in rabbit colonic SMC

**Figure 5:**
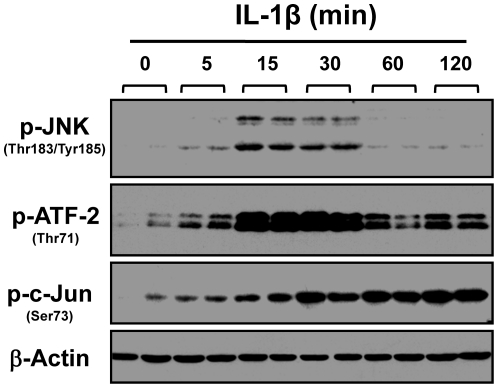
IL-1β induces a rapid and transient phosphorylation of JNK and sustained phosphorylation of ATF-2 and c-Jun in rabbit colonic smooth muscle cells. Cultured and serum-starved muscle cells were treated with IL-1β (10 ng/ml) for the indicated time period, followed by Western blot analysis with indicated anti-phospho antibodies. The β-actin was used for the loading control.

The data from pharmacological inhibition, gene reporter assay, mutagenic analysis, EMSA and CHIP assay suggest that JNK-AP1 pathway is activated when rabbit colonic SMC were exposed to IL-1β. To provide further experimental evidence, we performed Western blot analysis using phosphor-specific antibodies against the key members of JNK pathway. IL-1β treatment induced a rapid and transient increase in the phosphorylation of JNK at Thr-183/Tyr-185 (Fig. 5). ATF-2 and c-Jun are the major downstream substrates of JNK kinase and both bind to AP1 response elements in many other types of cells [40], [47]. Therefore, we determined the level of JNK-specific phosphorylation of ATF-2 at Thr-71 and c-Jun at Ser-73 in rabbit colonic SMC. As shown in Fig. 5, IL-1β stimulation induced rapid and sustained phosphorylation of both ATF-2(Thr-71) and c-Jun(Ser-73), implying the activation of ATF-2 and c-Jun by IL-1β-stimulated JNK pathway.

### MEKK1-MKK4 overexpression inhibited the constitutive and IL-1β-induced expression of Rgs4 protein

**Figure 6:**
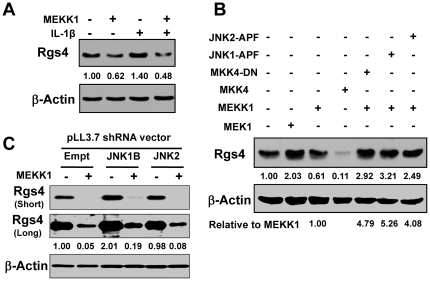
MEKK1-MKK4-JNK pathway inhibits Rgs4 expression in rabbit colonic smooth muscle cells. ***A. Overexpression of MEKK1 inhibited constitutive and IL-1β-induced Rgs4 expression.*** Cultured muscle cells were transfected with pCMV empty vector or MEKK1 vector for 24 h. After serum starvation for 24 h, cells were treated with or without IL-1β (10 ng/ml) for 3 h before Western blot analysis with anti-Rgs4 antibody. The β-actin was used for the loading control. The number between each blot indicates the relative fold of optical density compared to the corresponding control. ***B. Inhibitory effect of MEKK1 on RGS4 expression was blocked by the dominant mutants of MKK4, JNK1 and JNK2.*** Cells were cotransfected with indicated vectors for 24 h. After serum starvation for 24 h, Western blot analysis with anti-Rgs4 and anti-β-actin antibodies was performed. The number between each blot indicates the relative fold of optical density compared with the pCMV empty control. The number below the lower blot indicates the fold change related to MEKK1 group. ***C. Inhibitory effect of MEKK1 on RGS4 expression was partially reversed by the shRNA of JNK1B and JNK2A.*** Cells were cotransfected with indicated vectors for 48 h, followed by 24 h serum-starvation and Western blot analysis with anti-Rgs4 and anti-β-actin antibodies. Short (5 second) and longer (5 minute) exposures are presented. The number between each blot indicates the relative fold of optical density compared with the pCMV empty control.

MEKK1 is the key upstream kinase of JNK and induces dual phosphorylation of Thr/Tyr residues within a Thr-Pro-Tyr motif of JNK via the dual specific kinases MKK4 (also known as SEK1 or MEK4) and MKK7 (SEK2) [40], [53], [55]–[57]. To address whether MEKK1 regulates Rgs4 expression, MEKK1 was overexpressed in SMC. MEKK1 overexpression inhibited the constitutive and IL-1β-induced expression of Rgs4 protein, which was reversed by coexpressing dominant-negative JNK1 and JNK2 mutants (Fig. 6A, B) as well as JNK1 and JNK2 shRNA (Fig. 6C). Consistently, overexpression of MKK4 inhibited the constitutive Rgs4 expression, while overexpression of MKK4 dominant-negative mutant (MKK4-DN) blocked MEKK1-induced inhibition of Rgs4 expression (Fig. 6B), implying that MKK4 acts downstream of MEKK1 [53], [55], [58] and negatively regulates Rgs4 expression. In contrast, overexpression of MEK1, the key upstream kinase of ERK pathway, increased the constitutive expression of Rgs4 (Fig. 6B), which is consistent with our previous report showing that MEK1/ERK inhibition blocked IL-1β-induced upregulation of Rgs4 expression [39]. These data suggest that MEKK1-MKK4-JNK pathway harnesses inhibitory effect on Rgs4 expression in colonic SMC.

### JNK pathway interacts with p38 MAPK and NFκB pathways

**Figure 7:**
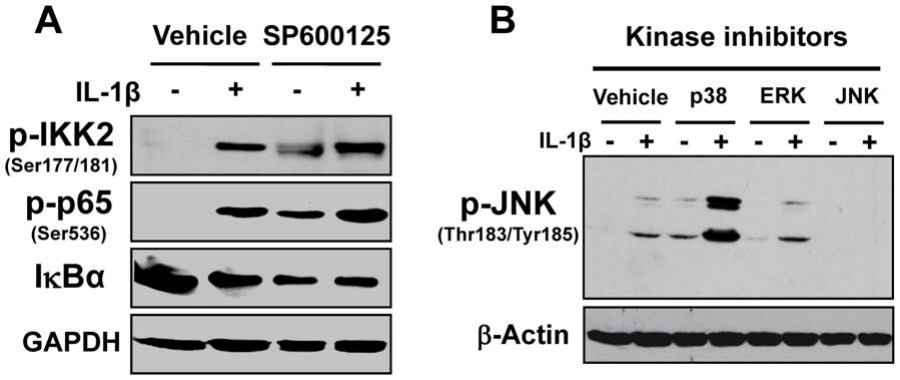
JNK pathway interacts with NFκB and p38 MAPK pathways. ***A. SP600125 enhances IL-1β-induced activation of canonical IKK2/IκBα/NFκB signaling.*** Cultured muscle cells after serum starvation for 24 h were pretreated with vehicle DMSO or JNK inhibitor SP600125 (10 µM) for 1 h before treatment with or without IL-1β (10 ng/ml) for 15 min. Activation of NFκB signaling was determined by Western blot analysis using indicated specific antibodies. ***B. IL-1β-induced phosphorylation of JNK (Thr183/Tyr185) is blocked by SP600125, enhanced by p38 MAPK inhibitor but not affected by MEK1 inhibitor.*** Cultured and serum-starved muscle cells were pretreated with p38 MAPK inhibitor SB203580 (1 µM) or MEK1 inhibitor PD98059 (20 µM) or JNK inhibitor SP600125 for 1 h before exposure to IL-1β (10 ng/ml) for 15 min. Activation of JNK pathway was determined by Western blot analysis using anti-phospho JNK antibody. The antibodies against GAPDH and β-actin were used for the loading control.

**Figure 8:**
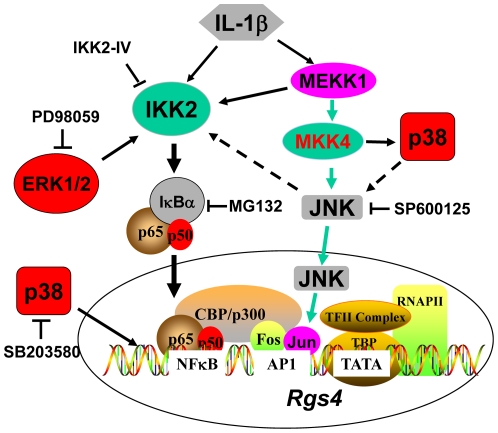
Schematic model for IL-1β-induced upregulation of Rgs4 expression in colonic smooth muscle cells via canonical IKK2/IκBα/NFκB signaling differentially modulated by MAPK pathways. IL-1β induces NFκB activation involving phosphorylation of IKK2, degradation of IκBα and nuclear translocation of p65/p50 leading to upregulation of *Rgs4* mRNA expression. IL-1β also activates three MAPKs. ERK1/2 and p38 MAPK enhance while JNK inhibits IL-1β-induced *Rgs4* upregulation. The effect of ERK1/2 is exerted on the canonical IKK2/IκBα/p65 pathway of NFκB activation and p38 MAPK may target at the chromatin level. The p38 may also inhibit JNK activity. Activation of the MEKK1-MKK4-JNK pathway down-regulates *Rgs4* expression through transcriptional repression at the chromatin level (via AP1 binding) and also signal inhibition of NFκB activation at the level of IKK2. The intricate interactions across various transcription factors and chromatin remodeling need further investigation. The solid arrows indicate the activation while the spotted arrows represent the inhibition.

Our previous studies have shown that the canonical IKK2/IκBα pathway of NFκB activation mediates IL-1β-induced upregulation of Rgs4 [6] and such upregulation is enhanced by the activation of the ERK1/2 pathway [39]. However, the stimulatory effect of p38 MAPK pathway on Rgs4 expression is independent of NFκB signaling [39]. To determine if NFκB, p38 MAPK and ERK1/2 pathways are involved in the JNK-AP1 pathway, we performed Western blot analysis in rabbit colonic SMC treated with selected MAPK inhibitors. The treatment with the JNK specific inhibitor (SP600025, 10 µM) alone induced a constitutive activation of NFκB signaling as determined by the phosphorylation of IKK2(Ser1-77/181) and p65 (Ser-536) as well as the degradation of IκBα [6]. Pretreatment with SP600125 1 h before IL-1β exposure enhanced IL-1β-induced NFκB activation (Fig. 7A). Treatment with the p38 MAPK inhibitor (SB203580, 1 µM) increased the constitutive and IL-1β-induced phosphorylation of JNK at Thr-183/Tyr-185 (Fig. 7B). However, the MEK inhibitor (PD98059, 20 µM) had no effect on the constitutive and IL-1β-stimulated phosphorylation of JNK at Thr-183/Tyr-185. The specificity of IL-1β-induced JNK phosphorylation was validated by the complete blockade with the JNK specific inhibitor SP600125 (10 µM). These data suggest that JNK activation inhibits NFκB signaling at the level of IKK2, which may also contribute to the tonic inhibition of JNK pathway on Rgs4 expression, and p38 MAPK negatively regulates JNK activity (Fig. 8).

## Discussion

The salient finding of this study is the identification of the tonic inhibitory regulation of *Rgs4* transcription by the activation of MEKK1-MKK4-JNK-AP1 signaling pathway. In a series of previous studies, we demonstrated that pro-inflammatory cytokine IL-1β upregulates Rgs4 expression in rabbit colonic SMC [7] through the canonical IKK2/IκBα pathway of NFκB activation [6] as well as ERK1/2 and p38 MAPK pathways [39]. This upregulation of Rgs4 is negatively regulated by the activation of PI3K/Akt/GSK3β pathway [39]. Here, we demonstrate an additional signaling pathway MEKK1-MKK4-JNK-AP1 that maintains a tonic inhibitory regulation on *Rgs4* transcription. The positive and negative regulatory mechanisms of Rgs4 expression reflect an intricate and delicate system for gene regulation (Fig. 8).

Rgs4 is implicated in intestinal inflammation [6], [7], [59], [60], cardiovascular diseases [61]–[63] and psychiatric disorders [4], [8]–[12]. However, the regulatory mechanism of Rgs4 expression has not been well understood. We and others have demonstrated that *Rgs4* expression is transcriptionally regulated [6], [38], [64]–[67]. We have cloned and characterized the promoter region of rabbit *Rgs4* [38]. This promoter contains a canonical TATA box, and predicted binding sites for several transcription factors such as NFκB, AP1, GATA, MyoD, etc. Similar promoter regions have been identified in human [65]–[67], rat [64] and mouse [65] *Rgs4*. Within human *RGS4* promoter, the inverted CCAAT box element (ICE) and the cAMP response element (CRE) mediate activation while the B-cell lymphoma 6 (Bcl6)-binding site mediates repression of *RGS4* transcription [67]. Within rat *Rgs4* promoter, a variant AP1-related site mediates transcriptional repression [64]. For mouse *Rgs4* promoter, no experimental evidence for the functional regulation has been reported [65]. For rabbit *Rgs4* promoter, we have identified the important role of NFκB binding site in mediating IL-1β-induced upregulation of *Rgs4* mRNA expression [6]. In the present study, we validated the AP1 binding site within the proximal region of rabbit *Rgs4* promoter using *ex vivo* CHIP, *in vitro* EMSA and site-directed mutagenic analysis. The AP1-DNA binding activity was significantly increased by IL-1β treatment in rabbit colonic SMC. Western blot analysis demonstrated a rapid activation of the JNK-AP1 pathway by IL-1β. The activation of the JNK-AP1 pathway induced a tonic repression of *Rgs4* transcription. The following evidence supports our findings: (1) Either specific inhibition of JNK with SP600125 or mutation of the proximal AP1 binding site within rabbit *Rgs4* promoter significantly increased the basal and IL-1β-inducible promoter activity; (2) Specific inhibition of JNK with SP600125 and shRNA increased the basal level of Rgs4 expression and potentiated IL-1β-induced upregulation of Rgs4 expression; (3) Overexpression of MEKK1/MKK4 inhibited Rgs4 expression while overexpression of MKK4/JNK mutants and JNK shRNA reversed MEKK1-mediated Rgs4 inhibition.

The family of MAPKs (all members) is activated upon dual phosphorylation at threonine and tyrosine by upstream kinases in response to diverse extracellular stimuli. However, the role and outcome of the activation of MAPK pathways rely on the stimuli, target genes and cell resources. The selective involvement of an individual MAPK pathway can be identified generally by specific manipulation of each pathway. In most cases, the MAPK pathways mediate the upregulation of many target genes including inflammatory mediators, contractile proteins and signaling components/regulators. In airway SMC, IL-1β-induced upregulation of COX-2 and eotaxin is inhibited by either MEK1 inhibitors or p38 MAPK inhibitors [68]–[70], whereas IL-1β-induced RANTES release is sensitive to inhibition of MEK1 [71] or JNK [72] but not inhibition of p38 MAPK [71]. IL-1β-induced upregulation of MMP-9 [48] and tumor necrosis factor α-induced expression of VCAM-1 [73] are sensitive to the inhibition of all three MAPK pathways. In vascular SMC, IL-1β-stimulated iNOS expression is prevented by MEK1 inhibition but potentiated by p38 MAPK inhibition [74], [75]. Inhibition of MEK1 or p38 MAPK, but not PI3K, reduced IL-1β-stimulated expression of LIMK2 and cofilin [76]. However, in human vascular SMC, IL-1β activates only p38 MAPK, which mediates IL-1β-induced IL-8 and VEGF expression [77], [78]. In human colonic SMC, IL-1β-induced H_2_O_2_ production is inhibited by MEK inhibitor but not p38 MAPK inhibitor [79], while IL-1β-induced upregulation of IL-6, IL-8, and COX-2 is reduced by p38 MAPK inhibitor but not MEK-1 inhibitor [80]. In rabbit colonic SMC, IL-1β-induced upregulation of Rgs4 is attenuated by MEK and p38 MAPK inhibitors but is potentiated by PI3K inhibitors [39]. The present studies demonstrate for the first time that JNK inhibitor and shRNA potentiate the constitutive and inducible expression of Rgs4 in rabbit colonic SMC.

In our previous studies, we showed that IL-1β consistently induced a 10–20 fold increase in mRNA expression of endogenous *Rgs4* in colonic SMC [6], [7]. However, reporter gene assay using *Rgs4* promoter detected only a 1–2 fold induction by IL-1β in rabbit colonic SMC [38]. Weak induction in the reporter gene assay also occurred as to the stimulatory effect of SP600125 (Fig. 2). Such discrepancy may be interpreted as the following: (i) IL-1β-induced upregulation of endogenous *Rgs4* mRNA level involves not only the transcriptional mechanism but also other mechanisms such as HuR-mediated mRNA stability [81]; (ii) The constitutive promoter activity without IL-1β treatment is already high, which may limit further induction; (iii) The promoter used contains only the proximal region, not reflecting the true full-length functional promoter of *Rgs4* [38]; and (iv) JNK pathway may regulate the endogenous *Rgs4* through other signaling pathways not related to the promoter region. The JNK-AP1 pathway has been shown to regulate mRNA stability of many genes through down-regulating the expression of HuR [82], [83] or upregulating tristetraprolin [84].

The mechanism underlying the inhibition of JNK-AP1 pathway on *Rgs4* transcription remains to be determined. In rat *Rgs4* promoter, FRA-2-dependent dismissal of the transcriptional co-activator, CRE-binding protein is involved in AP1-mediated transcriptional repression [64]. In the present study, we demonstrated that IL-1β treatment induced the recruitment of both c-Fos and c-Jun but dismissed ATF-2 from the AP1-binding site of rabbit *Rgs4* promoter. Thus, different dimers of AP transcription factor may function in different ways. IL-1β induction may promote preferentially the binding of Fos/Jun heterodimer and/or Jun/Jun homodimer to the heptamer consensus sequence of AP1 site [TGA(C/G)TCA]. Such binding represses rabbit *Rgs4* transcription. In contrast, ATF-2-containing dimers may normally bind to the AP1 site and activate *Rgs4* transcription. Upon JNK activation by IL-1β induction, the ATF-2-containing activator was removed and Jun-containing repressor was strengthened, leading to tonic inhibition of *Rgs4* transcription. JNK1 and JNK2 have mostly overlapping functions due to their concurrent and ubiquitous expression, although recent evidence identified their opposing effects [85]. In the present study, the dominant-negative mutants and shRNA of JNK1 and JNK2 generate similar effect on Rgs4 protein expression. The stronger stimulation of Rgs4 expression by JNK2 shRNA may result from more efficient knockdown of JNK2, although the possibility of a distinct role between JNK1 and JNK2 cannot be ruled out [85]. Other members of Fos, Jun and ATF subfamily [54] as well as other JNK-regulated transcription factors (NFAT, SMAD) [45]–[47] may also contribute to the JNK-induced inhibition of Rgs4 expression.

Ubiquinylation and arginylation of Rgs4 lead several bands of Rgs4 on the Western blot [6], [34]–[36], [63]. Rgs4 protein is regulated by the N-end rule pathway [34], [35] and proteasome degradation [6], [36]. Our previous studies showed that proteasome inhibition by MG132 increases Rgs4 protein expression [6]. In the present study, we demonstrated that the protein bands and levels of Rgs4 are increased by both JNK inhibitor SP600125 and JNK1/JNK2 shRNA. This result suggests that JNK pathway may affect ubiquinylation and/or proteasome degradation of Rgs4, in addition to the transcriptional and posttranscriptional regulation. Further study is needed to validate whether and how JNK and other MAPK pathways regulate post-translational modification of Rgs4.

Previous studies have targeted the effects of these MAPK pathways on the proliferation, migration, differentiation and cell death of SMC [86]. However, the role of MAPK in regulating SMC contraction remains poorly understood. Recent evidence suggests that both ERK1/2 and p38 MAPK are implicated in the Ca^2+^ sensitization [87] and protein kinase C-dependent contraction of gastrointestinal smooth muscle [87]–[89]. Phosphorylation of caldesmon and/or calponin may contribute to the effect of ERK1/2 [90]–[92] and JNK [93], whereas p38 MAPK may regulate muscle contraction through sequential phosphorylation and activation of MAPKAPK-2 [94] and HSP27 [95], [96]. In airway SMC, both ERK1/2 and JNK, but not p38 pathway, are responsible for IL-1β-induced inhibition on the contractile response to endothelin receptor agonist [97]; JNK pathway also mediates Toll-like receptor-mediated airway hyper-responsiveness to bradykinin [98]. In vascular SMC, all three MAPK pathways are involved in the contractile signaling [93], [99]. In ileal SMC, sphingosyl phosphorylcholine-induced contraction is blocked by MEK-1 inhibitor but not p38 MAPK inhibitor [100]. In esophageal SMC, ERK1/2 but not p38 and JNK contributes to sphingosine 1-phosphate-induced contraction [15], [101] and bombesin-induced contraction [102]. However, all three MAPK pathways (p38, ERK1/2 and JNK) mediate LPS-induced inhibition on acetylcholine-stimulated contraction in rabbit duodenum containing SMC and enteric nervous system [51], [103], [104]. In animal colitis induced by 2,4,6-trinitrobenzene sulfonic acid, ERK1/2 mediates the restoration of the reduced muscle contractility by meloxicam, a COX2 inhibitor [105]. In dextran sulfate sodium-induced colitis, both ERK and p38 MAPK pathways contribute to hypercontractility but JNK was not studied [106]. The present study provides the first evidence that the JNK pathway maintains the low level of Rgs4 expression in colonic SMC and subsequently leads to the promotion of SMC contraction. The tonic inhibition of Rgs4 expression by JNK pathway provides a new mechanism for the contribution of JNK pathway in regulating smooth muscle contraction [93], [98].

The cross-talk between JNK pathway and other MAPK and NFκB pathways is not well understood. The ERK1/2 pathway has been widely shown to affect IL-1β-induced NFκB activation and regulate Rgs4 expression [39]. The p38 MAPK pathway stimulates Rgs4 expression independently of NFκB signaling [39]. In the present study, we showed that p38 MAPK negatively regulates JNK activity but ERK1/2 pathway does not affect JNK pathway. However, the JNK and NFκB pathways regulate each other during IL-1β-induced upregulation of Rgs4 expression in rabbit colonic SMC. JNK activation inhibits NFκB signaling at the level of IKK2. To the contrary, IKK2-mediated NFκB signaling promotes IL-1β-induced activation of the JNK-AP1 pathway because IKK2 inhibitor abolished IL-1β-stimulated AP1-binding activity within Rgs4 promoter. Our conclusion is supported by several previous studies showing a positive regulation of JNK pathway by IKK [107]–[109]. The mechanism underlying IKK2-mediated activation of JNK pathway remains to be determined.

In conclusion, activation of MEKK1-MKK4-JNK-AP1 signaling pathway plays a tonic inhibitory role in regulating *Rgs4* transcription in rabbit colonic SMC. Rgs4 expression is dynamically and strictly regulated by both the positive signaling pathways of NFκB, ERK1/2 and p38 MAPK and the negative pathways of PI3K-Akt-GSK3β and MEKK1-MKK4-JNK-AP1. This intricate and orchestral regulation may aid in maintaining the transient function of Rgs4 for smooth muscle contraction/relaxation as well as cardiovascular and neuronal functions.

## Materials and Methods

### Reagents and antibodies

IL-1β was obtained from Alexis Biochemicals (San Diego, CA). SP600125 (Anthra[1,9-cd]pyrazol-6(2H)-one, 1,9-pyrazoloanthrone), PD98059 (2′-Amino-3′-methoxyflavone), SB203580 (4-(4-Fluorophenyl)-2-(4-methylsulfinylphenyl)-5-(4-pyridyl)1H-imidazole), and IKK2-IV (IKK2 inhibitor IV, [5-(p-Fluorophenyl)-2-ureido]thiophene-3-carboxamide) were obtained from EMD Chemicals (San Diego, CA) and dissolved in dimethyl sulfoxide (DMSO). Antibodies against c-Fos, c-Jun, ATF-2, JNK(FL), IκBα, GAPDH and β-actin were obtained from Santa Cruz Biotechnology (Santa Cruz, CA). Affinity-purified anti-Rgs4 antibody was kindly provided by Dr. Susanne M. Mumby (University of Texas Southwest Medical Center). Antibodies against phospho-JNK(Thr183/Tyr185), phospho-ATF-2(Thr71), phospho-c-Jun(Ser73), phospho-IKK2 (Ser177/181), phospho-IκBα (Ser32/36) and phospho-p65 (Ser546) were from Cell Signal Technology (Danvers, MA). All the other reagents were from Sigma (St. Louis, MO).

### Ethics Statement

All procedures involving rabbit were approved by the IACUC committee at Temple University (approval protocol # 3164) or Virginia Commonwealth University (approval protocol # 0510-3402).

### Isolation and culture of SMC

Rabbit colonic circular muscle cells were isolated and cultured as previously described [7]. Briefly, distal colon from euthanized New Zealand White rabbits (2∼2.5 kg) was placed in HEPES-buffered smooth muscle media. The circular smooth muscle layer was dissected from the mucosa and longitudinal muscle layer using stereo microscopy and treated with 0.1% collagenase (type II) and 0.1% soybean trypsin inhibitor for 30 min at 31°C. The isolated single muscle cells were harvested after several rounds of spontaneous dispersion by filtration through 500-µm Nitex and centrifuged twice at 350 g for 10 min. The isolated SMC were cultured in 100 mm dish with DMEM containing 10% fetal bovine serum and 1% antibiotics and antimycotics. After 10–14 days, the SMC attained confluence and were then passaged once for use in various experiments. Full confluent muscle cells were deprived of serum for 24 h before experiments.

### Promoter cloning, site-directed mutagenesis and vector construction

The rabbit *Rgs4* promoter containing a fragment of −962/+50 (from the putative transcription start site) was cloned into pMlu3 AccepTor vector as described previously. The potential binding site for AP1 transcription factor was identified by MatInspector (http://www.genomatix.de) and TFSEARCH (http://www.cbrc.jp) and located at −213/−203 of rabbit *Rgs4* promoter as previously described [38]. Mutation of the AP1 binding site (ATTGAGTCACT) in the pMluc3-*Rgs4*-P2 reporter vector construct was performed by site-directed mutagenesis using the QuikChange kit (Stratagene). Mutagenic primers (sense, 5′-GACATTTGTAGAGATGACATCAGTCGTTTTTCATGTATG-3′ and anti-sense 5′-CATACATGAAAAACGACTGATGTCATCTCTACAAATGTC-3′) led to a nucleotide change in the entire binding site for AP1 transcriptional factor. Mutation was confirmed by nucleotide sequencing.

Mammalian expression vectors encoding MEK1 and MEKK1 were obtained from Clontech. Mammalian expression vectors encoding MKK4, MKK4-DN, JNK1-APF and JNK2-APF were generously provided by Dr. Riches J. David (National Jewish Center, Denver, CO) [53], [55].

The JNK shRNA expression vectors were generated as previously described [110]. JNKs originate from three genes that yield 10 isoforms through alternative mRNA splicing. Since colonic SMC expresses JNK1 and JNK2, we designed two shRNA-encoding sequences for JNK1 and JNK2. The JNK1A and JNK1B shRNA targeted the nucleotides 124–149 and 339–360 of rabbit JNK1 (XM_002722671). The JNK2A and JNK2B shRNA targeted the nucleotides 647–699 and 747–771 of rabbit JNK2 (XM_002721308.1). The shRNA expression cassette was generated through consequential, two rounds of PCR, and cloned into pLL3.7 lentiviral vector which contains CMV-promoted EGFP (enhanced green fluorescent protein) marker as an internal control [110]. The sequence of each shRNA expression cassette in the vector was confirmed by restriction enzyme digestion and DNA sequencing.

### Cell transfection and reporter Assays

All the mammalian expression vectors were prepared using EndoFree Plasmid Maxi kit (Qiagen). All transfections in rabbit colonic SMCs were performed utilizing a Lipofectamine-2000 kit (Invitrogen) as previously validated [6], [39], [110]. The transfection efficiency of rabbit SMC (∼60%) was determined by the expression of internal EGFP in the pLL3.7 shRNA expression vector. For Western blot analysis, cells (5×10^5^/well) cultured in a 6-well plate were cotransfected with indicated vectors for 24–48 h followed by serum starvation and treatment. For reporter assays, cells (2–4×10^4^/well) cultured on a 96-well plate were cotransfected with the *renilla* luciferase reporter constructs and the 1∶10 normalization *firefly* luciferase vector pGL4-CMV (Promega).

After incubation with IL-1β for 24 h in the absence or presence of JNK inhibitor SP600125, the media were harvested for measurement of *renilla* luciferase activity and the cell lysate was used for measurement of *firefly* luciferase activity. The *renilla* luciferase was determined with a *renilla* luciferase assay kit (Promega). The *firefly* luciferase was determined using a ONE-Glo luciferase assay system (Promega). The luminescence was measured using EnVision multilabel plate reader (Perkin Elmer). Data are normalized by dividing *renilla* luciferase activity with that of the corresponding *firefly* luciferase activity. Four to six separate experiments were conducted anddata was calculated in each experiment as the average of 4–6 samples.

### Reverse transcription (RT) quantitative PCR (RT-qPCR)

Cells were treated with the Trizol reagent (Invitrogen, Carlsbad, CA) for total RNA extraction. The potentially-contaminated genomic DNA was removed by treating 10 µg of the RNA sample at 37°C for 30 min with 1 µl of TURBO DNase (Ambion, Austin, TX) followed by extraction with phenol∶chloroform∶isoamylalcohol (25∶24∶1). Real time PCR analysis was carried out on the ABI Prism® 7300 Sequence Detection System (Applied Biosystems, Foster, CA). Expression of *Rgs4* was analyzed using the TaqMan® PCR Master Mix Reagents Kit (Applied Biosystems). The TaqMan probe and primers for rabbit *Rgs4* designed using the Primer Express® 2.0 version were as follows: (forward, nucleotides 232–252, exon 2) 5′-tcccacagcaagaaggacaaa-3′, (reverse, nucleotides 303–284, exon 3) 5′-ttcggcccatttcttgactt -3′ and (probe, nucleotides 254–279, across exon 2 and 3 with 321 bp of intron 2) 5′-ttgactcaccctctggcaaacaacca-3′. The cDNA was synthesized from 500 ng of RNA using the TaqMan® RT Reagents Kit (Applied Biosystems). The optimized concentrations for real-time PCR were 0.4 µM for both primers and 0.2 µM for the probe, and 5 ng cDNA in a 20 µl reaction volume. Rabbit GAPDH primers (forward 5′-cgcctggagaaagctgctaa-3′, reverse 5′-cgacctggtcctcggtgtag -3′) were used as an internal control. Each sample was tested in triplicate. Cycle threshold (Ct) values were obtained graphically for *Rgs4* and GAPDH. The difference in Ct values between GAPDH and *Rgs4* were represented as ΔCt values. The ΔΔCt values were obtained by subtracting the ΔCt values of the control samples from that of the treated samples. Relative fold change in gene expression was calculated as 2−ΔΔCt.

### Western blot analysis

Cells were solubilized for 30 min in Triton X-100-based lysis buffer containing 20 mM Tris (pH 7.5), 150 mM NaCl, 1% Triton X-100, 1 mM EDTA, 100 µg/ml phenylmethylsulfonyl fluoride, 10 µg/ml aprotinin, 10 µg/ml leupeptin, 30 mM sodium fluoride and 3 mM sodium vanadate. After centrifugation of the lysates at 20,000 g for 10 min at 4°C, the protein concentrations of the supernatant were determined with a Dc Protein Assay kit from BioRad (Hercules, CA). Equal amounts of protein were fractionated by SDS-polyacrylamide gel electrophoresis, and transferred to nitrocellulose membrane (BioRad). Blots were blocked in 5% nonfat dry milk/tris-buffered saline (pH 7.6) plus 0.1% Tween-20 (TBS-T) for 1 h and then incubated overnight at 4°C with various primary antibody in TBS-T plus 1% milk. The dilution of 1∶1000 was used for most primary antibodies except for anti-Rgs4 (1∶10,000) and β-actin (1∶100,000). After incubation for 1 h with horseradish peroxidase-conjugated corresponding secondary antibody (1/2,000; 10 µg/ml, Pierce) in TBS-T plus 1% milk, immunoreactive proteins were visualized using SuperSignal Femto maximum sensitivity substrate kit (Pierce, Rocjford, IL). All washing steps were performed with TBS-T.

### Electrophoretic Mobility Shift Assay (EMSA)

Rabbit colonic SMC were cultured into full confluency and starved with serum-free culture media for 24 h. Cells were pretreated with vehicle (DMSO), JNK inhibitor SP600125 or IKK2 inhibitor IKK2-IV for 1 h before treatment with IL-1β for 1 h. Nuclear extracts were prepared using NE-PER Nuclear and Cytoplasmic Extraction Reagent Kit (Pierce, Rockfold, IL). The oligonucleotide probe covering the predicted AP1 binding site within the promoter of rabbit *Rgs4* was used. Synthesized sense (5′- tcgaCATTTGTAGAGATATTGAGTCACTTT-3′) and antisense 5′-tcgaAAAGTGACTCAATATCTCTACAAATG-3′) oligonucleotides were annealed to generate a double-strand DNA probe with an overhang TCGA for end-labeling. The probe was labeled with γ-^32^P-ATP and T4 polynucleotide kinase (Promega), and added to the binding reactions in the presence of poly(dI-dC)∶poly(dI-dC) (Sigma), herring sperm DNA (Invitrogen, Carlsbad, CA), and nuclear extracts. Equal amounts of extracts (10 µg) were loaded for each binding reaction. After 30 min incubation at room temperature, samples were loaded onto a pre-electrophoresed 0.5× tris-borated EDTA buffer (TBE), 6% polyacrylamide gel and run at 150 V for approximately 1.5 h. The gels were then fixed and dried, and autoradiographs obtained.

### Chromatin immunoprecipitation (CHIP) assay

CHIP assay was performed according to the manufacture's protocol (Upstate Biotechnology Inc., Lake Placid, NY). Cells were cultured in 10-cm dishes until full confluence was established and then serum-starved overnight. Cells were treated with IL-1β (10 ng/ml) for 3 h. The DNA-chromatin of cells were cross-linked by the addition of 280 µl of 37% formaldehyde to 10 ml of culture medium for 10 min at room temperature and stopped with 0.125 M glycine. Cells were washed twice with PBS and harvested with 1 ml of SDS lysis buffer (20 mM Tris-HCl, pH 8.0, 140 mM NaCl, 1% Triton X-100, 1% SDS, 1% deoxycholic acid, 2 mM EDTA, and freshly added protease inhibitors). After sonication and centrifugation, the supernatants were used for standard immunoprecipitation with anti-c-Fos antibody or control IgG and protein A/G agarose bead (Santa Cruz). The immune complexes were eluted, reverse cross-linked using 5 M NaCl, and purified by phenol/chloroform extraction. Ethanol-precipitated DNA pellets were dissolved in Tris-EDTA buffer. The supernatant of an immunoprecipitation reaction carried out in the absence of antibody was purified and diluted 1∶100 as total input DNA control. PCR was carried out on 1 µl of each sample using sense and anti-sense primers against the cloned promoter region of rabbit *Rgs4*. PCR products were analyzed on 1% agarose gels and images were analyzed with NIH ImageJ densitometric measurements. Relative changes were calculated using the mean density after background subtraction.

### Statistical analysis

The images from Western blot, EMSA and CHIP assays were scanned and analyzed with NIH ImageJ (1.46a version) densitometric measurements. The data were expressed as integrated density and presented as relative fold in comparison with the corresponding control. Quantitative data were expressed as means ± SE of n experiments and statistical significance was determined using Student's *t*-test for unpaired values or ANOVA and Newman-Keuls comparison.
